# Comparing the Outcomes of Cast Immobilization with and Without K-Wire Fixation for Displaced Distal Radius Fractures in the Pediatric Population: A Systematic Review and Meta-Analysis

**DOI:** 10.3390/medicina61050852

**Published:** 2025-05-06

**Authors:** Muteb N. Alotaibi, Lamya Ghanim A. Aldaraani, Abdulaziz S. Altala, Aseel A. Alqurashi, Ismail S. Alateeq, Abdulkarim Abdullatif Alkhamisi, Ibrahim Saleh Allehaimeed, Ammar Yasser Jad, Hamed Alosaimi, Bassam H. Alharbi, Ahmad Alenezi, Aliyah Zayed Almutairi, Fares Khalid Alroudhan, Mahdi Mofarah Alqarni

**Affiliations:** 1 College of Medicine, Alfaisal University, Riyadh 13523, Saudi Arabia; 2 Ministry of Health, Riyadh 62521, Saudi Arabia; lamya.ald98@gmail.com; 3 College of Medicine, Dar Aluloom University, Riyadh 13523, Saudi Arabia; 1192490@du.edu.sa (A.S.A.); k.fares.k@hotmail.com (F.K.A.); 4 College of Applied Medical Science, Al Taif University, Taif 26511, Saudi Arabia; aseelalqurashi78@gmail.com; 5 College of Medicine, Imam Mohammad Ibn Saud Islamic University, Riyadh 13523, Saudi Arabia; ismailoresearch@gmail.com; 6 College of Medicine, King Abdulaziz University, Jeddah 22233, Saudi Arabia; alkhamisiabdulkarim@gmail.com (A.A.A.); bassam.alharbi171@gmail.com (B.H.A.); 7 General Practice, Qassim Health Cluster, Buraydah 52261, Saudi Arabia; is.haimeed@gmail.com; 8 Ministry of Health, Jeddah 22233, Saudi Arabia; ammaryj7@gmail.com; 9 Ministry of Health, Buraydah 52261, Saudi Arabia; ihamed.hao@gmail.com; 10 Ministry of Health, Kuwait City 91104, Kuwait; dr.ahmadalenezi09@gmail.com; 11 College of Applied Medicine Sciences, King Saud University, Riyadh 13523, Saudi Arabia; aliyahalmotairi@gmail.com; 12 Department of Pediatric Orthopedics, Maternity and Children Hospital, Abha 62521, Saudi Arabia; dralqarnim@gmail.com

**Keywords:** cast immobilization, K-wire fixation, radius fractures, pediatric, systematic review

## Abstract

*Background and Objectives:* Distal radius fractures are among the most common pediatric injuries, accounting for approximately 25% of all fractures in children. Displaced fractures are prone to re-displacement, necessitating additional interventions. K-wire fixation is effective in reducing re-displacement risks, but no one has systematically assessed its use. This study aims to compare the outcomes of cast immobilization alone versus cast immobilization with K-wire fixation in pediatric patients with displaced distal radius fractures. *Methods:* A comprehensive search of PubMed, Web of Science, Cochrane CENTRAL, Scopus, and Embase databases for studies comparing these treatments. The quality assessment was conducted using the Cochrane Collaboration Risk of Bias for randomized studies and the Methodological Index for non-randomized studies. The meta-analysis was carried out using RevMan software V5.4. *Results:* Out of 267 initial records, 12 studies met the inclusion criteria, encompassing 1455 patients (853 treated with cast alone and 602 with K-wire fixation). Meta-analysis of 10 studies showed significantly higher re-displacement rates with cast immobilization compared to K-wire fixation (OR: 11.42, 95% CI: 2.43–53.77, *p* = 0.002, I^2^ = 82%). The risk of secondary surgery was also higher in the cast group (OR: 6.91, 95% CI: 1.5–31.72, *p* = 0.01, I^2^ = 75%). However, complications were lower with cast immobilization (OR: 0.68, 95% CI: 0.45–1.03, *p* = 0.07, I^2^ = 74%), though not statistically significant. *Conclusions:* K-wire fixation appears to offer superior fracture stability and reduces the need for secondary surgeries compared to cast immobilization for displaced distal radius fractures in pediatric patients. However, both treatment modalities are associated with comparable complication rates, emphasizing the importance of individualized treatment planning.

## 1. Introduction

Distal radius fractures (DRFs) are the most common fractures in children [1], which account for 25–43% of all fractures [2]. These fractures often result from falls on an outstretched hand [3]. Due to their rapidly developing skeletal structure, children and adolescents are especially susceptible to distal radius fractures, necessitating optimal treatment strategies to ensure favorable long-term outcomes during growth and development [4]. They represent approximately 19.9–35.8% of all pediatric fractures and are commonly managed through reduction and cast immobilization (RCI) [3,4,5,6]. Up to 34% of distal radius fractures have been reported to re-displace early following reduction [7,8,9]. The factors contributing to re-displacement after an initially successful closed reduction remain not clearly defined [7]. To prevent re-displacement following initial reduction and reduce the need for secondary intervention, displaced distal radius fractures (DDRFs) can be managed with reduction followed by percutaneous K-wire fixation (KWF) prior to cast immobilization [1].

K-wire fixation is widely utilized in the management of displaced distal radius fractures due to its ability to provide stable fixation with minimal soft tissue disruption [10]. It is particularly effective in maintaining fracture reduction in unstable or extra-articular fractures, making it a preferred choice in certain patient populations [11]. K-wire fixation offers a straightforward, quick, and minimally invasive option that is both affordable and requires minimal surgical tools. However, because the fixation lacks rigidity, patients usually need to be immobilized in a plaster cast for a minimum of four weeks. If the wires are left exposed, there is a risk of infection, while burying them beneath the skin may necessitate a second procedure for removal. Furthermore, removing the wires can sometimes lead to fracture collapse, potentially causing deformity and reduced function [12].

Our study aims to compare the outcomes of cast immobilization alone versus cast immobilization with K-wire fixation in pediatric patients with displaced distal radius fractures.

## 2. Materials and Methods

This systematic review and meta-analysis were conducted in accordance with the Preferred Reporting Items for Systematic Review and Meta-Analysis (PRISMA) guidelines (Table S1). This review is registered on PROSPERO (ID: CRD42024558640) [13].

### 2.1. Search Strategy and Eligibility Criteria

A systematic search was performed across PubMed, Web of Science, Cochrane Central, Scopus, and Embase from inception to June 2024. The search strategy included the following keywords and their combinations using Boolean operators (AND, OR): “displaced distal radius fractures”, “pediatric fractures”, “Kirschner wire”, “cast immobilization”, “fracture re-displacement”, “orthopedic surgical procedures in children”, “displaced distal radius fracture”, “pediatric”, and “distal radius”. A reference list of the included studies was searched for additional studies. Also, we have searched for the grey literature to minimize the risk of publication bias and improve the comprehensiveness of the evidence base.

We included all clinical studies that contained information on the following: (1) pediatric patients with displaced distal radius fractures; (2) studies comparing reduction followed by cast immobilization alone versus reduction with additional K-wire fixation; (3) studies with outcomes measuring re-displacement, secondary reduction, range of motion, and complications; (4) studies with at least 50% bone width displacement or angulation and required manipulation; and (5) studies written in English. Any study that did meet the criteria was excluded.

### 2.2. Study Selection and Data Extraction

Two authors independently screened the studies by title and abstract. Then, they reviewed the full text of potential, including studies for more detailed assessment. Any conflicts were solved by consulting a third author. Then, the articles included were extracted and reviewed by the same two authors. The extraction encompassed essential data such as (1) Study Characteristics: author, year of publication, country, study design, sample size, follow-up duration, and inclusion/exclusion criteria. (2) Patient Characteristics: Pediatric population with a minimum of 50% bone width displacement or angulation necessitating manipulation. (3) Intervention used: reduction followed by cast immobilization alone versus reduction with additional K-wire fixation. (4) Outcome Measures: Rate of re-displacement, secondary reduction rate, range of motion, and complications. (5) Data Analysis: Comparative studies comprising randomized controlled trials and cohort studies. (6) Conclusion: The study’s findings, limitations, and overall recommendations based on the findings.

### 2.3. Risk of Bias

The researchers assessed the risk of bias and methodological quality for each included study using the appropriate tool based on the study design. For randomized controlled trials (RCTs), the Cochrane Collaboration Risk of Bias (ROB) was used. Two reviewers independently assessed the risk of bias for each RCT, resolving disagreements through discussion. The overall risk of bias for each RCT was judged as low, high, or unclear based on the assessment of individual parts. The Methodological Index for Non-Randomised Studies (MINORS) was used for non-randomized studies, and disagreements were resolved through discussion.

### 2.4. Parameters Evaluated

In this systematic review and meta-analysis, we assessed multiple clinically relevant parameters. The primary outcomes included the rate of fracture re-displacement and the need for secondary interventions, such as re-manipulation or surgical fixation. Secondary outcomes comprised radiological outcomes, including residual angulation, bone shortening, and quality of reduction, as well as functional outcomes, such as forearm range of motion (ROM), pronation and supination angles, and, where available, grip strength. Additionally, we evaluated the incidence of complications, including malunion, infection, and pain.

### 2.5. Statistical Analysis

The statistical analysis for this study was conducted using standard statistical software, including RevMan and Excel, to calculate frequencies, percentages, means, and standard deviations for various clinical and demographic variables. RevMan was specifically utilized for meta-analysis calculations, generating forest plots, and assessing heterogeneity (I^2^), while Excel was employed for descriptive statistics.

Frequencies and percentages were used to describe categorical variables, such as gender, study design, and country of origin. Continuous variables, such as age, follow-up duration, and the number of patients in each group (cast immobilization alone and cast immobilization with K-wire fixation), were summarized using means and standard deviations (SD) for normally distributed data, or medians and interquartile ranges (IQR) for non-normally distributed data. Percentages were calculated by dividing the number of occurrences in each category by the total number of patients in each group and then multiplying by 100.

For continuous variables, mean values were calculated as the sum of all values divided by the number of observations, with standard deviation computed to assess variability around the mean. RevMan was employed to perform meta-analyses, calculating pooled odds ratios (OR) with 95% confidence intervals (CI) for outcomes such as re-displacement, secondary interventions, and complications. All the outcomes were assessed using the final measurements. Heterogeneity was assessed using the I^2^ statistic, and statistical significance was set at a *p*-value < 0.05. Any *p*-value below this threshold was considered statistically significant. To assess the risk of publication bias, Egger’s test was performed using R Studio version 4.4.2, and a *p*-value < 0.05 was considered indicative of potential publication bias. 

## 3. Results

### 3.1. Study Selection and Characteristics

**Figure 1:**
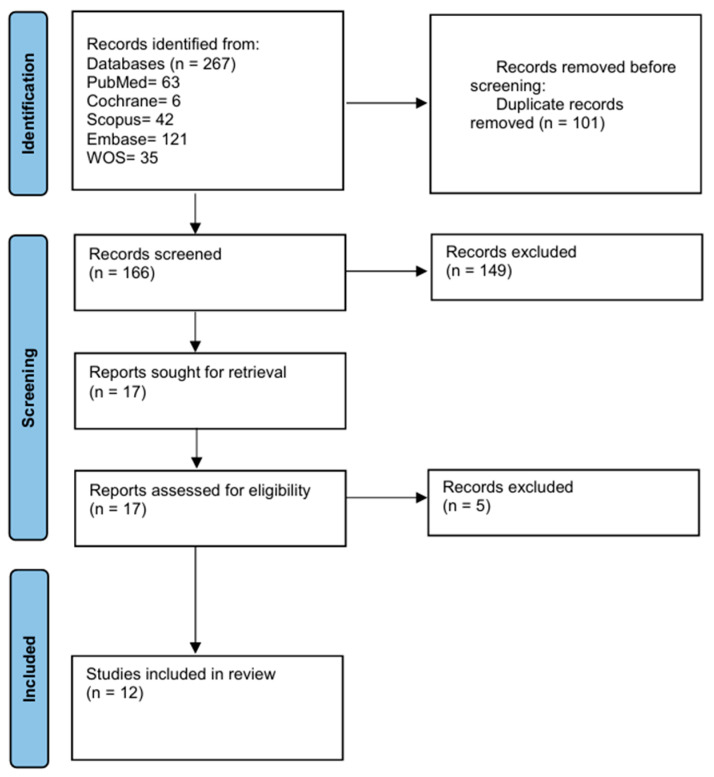
PRISMA flowchart illustrating the study selection process.

The literature search was conducted across five databases: Web of Science, Cochrane Central, Scopus, PubMed, and EMBASE, identifying a total of 267 studies (63 from PubMed, 121 from EMBASE, 35 from Web of Science, six from Cochrane Central, and 42 from Scopus). After removing 101 duplicates, 166 studies remained for eligibility screening. Subsequently, 148 studies were excluded, leaving 17 for full-text review. Ultimately, 12 studies published between 1994 and 2021 were included in the qualitative synthesis. The PRISMA flow diagram summarizes the study selection process (Figure 1).

A total of 12 studies were included in this systematic review and meta-analysis [14,15,16,17,18,19,20,21,22,23,24,25]. Among these studies, 6 were retrospective studies, 4 were randomized controlled trials (RCTs), and 2 were prospective randomized studies. The 12 studies originated from various countries, with the Netherlands contributing the most studies (5), followed by single studies from Germany, the United Kingdom, Scotland, Turkey, England, the United States, Belgium, and Switzerland, each contributing 1 study. Table 1 provides all the characteristics and patient-related demographics of all the studies included. Table S2 provides the inclusion and exclusion criteria for each study.

### 3.2. Patient Demographics and Baseline Characteristics

The 12 included studies assessed a variety of clinical and radiological outcomes related to the management of displaced distal radius fractures (DDRFs) with cast immobilization alone (Group 1) and cast immobilization with K-wire fixation (Group 2). The 12 studies included 1455 patients (ranging from 23 to 393 patients from each study). Of these, 853 patients received cast immobilization alone, while 602 patients underwent cast immobilization with K-wire fixation. The combined mean age across the studies is 9.86 years, with a pooled standard deviation (SD) of ±3.08 years. The patients’ ages ranged from 0 to 18 years, and the mean ages in the studies varied from 7.9 to 12.4 years, with standard deviations reported in some cases. Most studies included pediatric patients under 16 years old, with the highest mean age in a study focused on patients aged 10 to 14. Across 9 studies, there were 663 male patients (65.8%) and 345 female patients (34.2%). The mean treatment duration was 15.33 weeks (SD ±31.35 weeks), while the mean follow-up period was 22.4 weeks (5.6 months) with an SD of ±18.1 weeks. Follow-up times ranged from short-term (4–6 weeks) to long-term (up to 5 years), reflecting significant variability.

### 3.3. Clinical and Functional Outcomes by Meta-Analysis

#### 3.3.1. Intervention Details

Most patients in both the cast immobilization and cast immobilization and K-wire fixation groups presented with displaced distal radius fractures. Displaced fractures were reported in 9 studies for both treatment groups. Complete displaced fractures, including diaphyseal and metaphyseal fractures, were observed in 6 studies, while two studies included both displaced and non-displaced distal forearm fractures. Complete displaced diaphyseal fractures were specifically noted in 2 studies.

#### 3.3.2. Re-Displacement

**Figure 2:**
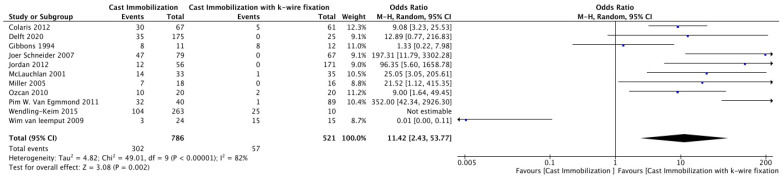
Forest Plot Comparing Re-Displacement Rates Between Cast Immobilization and K-Wire Fixation [14,15,16,17,18,19,20,21,22,24,25].

A meta-analysis of 11 studies comparing cast immobilization with K-wire fixation showed significantly higher re-displacement rates in the cast group (302/786) compared to the K-wire group (57/521) (Figure 2). The odds ratio (OR) was 11.42 (95% CI: 2.43–53.77, *p* = 0.002), indicating a substantially higher risk of re-displacement with cast immobilization. Heterogeneity was high (I^2^ = 82%, *p* < 0.00001), reflecting variability among studies. Some studies, such as Wendling-Keim 2015 [25] (OR: 352.00, 95% CI: 42.34–2926.30), reported extremely elevated odds for re-displacement with cast immobilization, while others, such as Wim van Iemput 2009 [15], had non-estimable odds ratios due to low event rates (Figure 2). Egger’s test showed significant evidence of publication bias among the included studies (*p* = 0.02).

#### 3.3.3. Secondary Interventions

**Figure 3:**
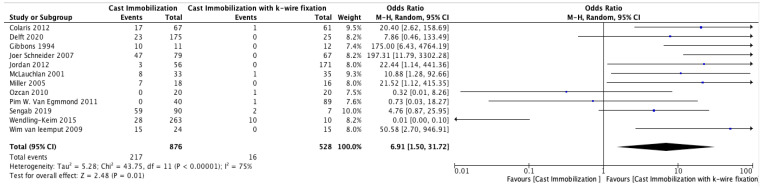
Forest Plot Comparing the Need for Secondary Surgery Between Cast Immobilization and K-Wire Fixation [14,15,16,17,18,19,20,21,22,23,24,25].

The meta-analysis comparing the need for secondary surgery between cast immobilization and K-wire fixation across 10 studies is shown in Figure 3. The overall odds ratio (OR) was 6.91 (95% CI: 1.5–31.72, *p* = 0.01), indicating a significantly higher risk of secondary surgery in the cast immobilization group. The heterogeneity was substantial (I^2^ = 75%, *p* < 0.00001), suggesting variability across the studies. Egger’s test showed significant evidence of publication bias among the included studies (*p* = 0.01).

#### 3.3.4. Complications

**Figure 4:**
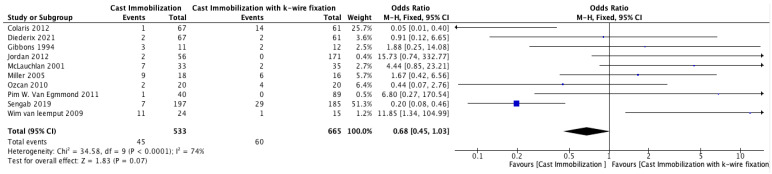
Forest Plot Comparing Complication Rates Between Cast Immobilization and K-Wire Fixation [14,15,16,17,18,19,21,22,23,24].

The meta-analysis comparing complication rates between cast immobilization and K-wire fixation across 10 studies is summarized in Figure 4. The overall odds ratio (OR) was 0.68 (95% CI: 0.45–1.03, *p* = 0.07), indicating an insignificantly lower risk of complications in the cast immobilization group compared to K-wire fixation. Heterogeneity was substantial (I^2^ = 74%, *p* < 0.00001), reflecting variability in the study outcomes. Egger’s test showed significant evidence of publication bias among the included studies (*p* = 0.04).

### 3.4. Systematic Review

#### 3.4.1. Radiological Outcomes

Radiological outcomes were reported in 6 of the 12 studies. One study found less residual angulation of the radius in the coronal plane in the casting group compared to the K-wire group, while another reported better quality of reduction and reduced need for X-ray follow-up in the K-wire group. Further loss of position during immobilization was observed in the casting group in one study. Radius bone shortening ranged from 3 to 14 mm in the cast group and 4 to 17 mm in the K-wire group, with one study reporting a mean shortening of 6.9 mm in the cast group versus 16.4 mm in the K-wire group.

#### 3.4.2. Range of Motion (ROM) Outcomes

ROM was assessed in 5 studies. Most studies found no significant differences in wrist ROM between the cast immobilization and K-wire fixation groups at both short- and long-term follow-up. In one study, the cast group showed a mean limitation of 5° (±11°), while the K-wire group had a limitation in prosupination of 5° (±8°). Another study found a higher loss of pronation in the cast group, though the difference was not statistically significant overall. Another study reported the mean limitation of pronation and supination as 14.3° (±13.6°) in the cast group and 6.9° (±9.4°) in the K-wire group.

#### 3.4.3. Angulation Outcomes

Angulation was reported in 5 of the 12 studies. One study reported a mean angulation of 25° (±14°) in the cast group and 23° (±18°) in the K-wire group. Another study found a significant difference, with the cast group showing a mean angulation of 9.1° (±11.5°) compared to 2.7° (±7.3°) in the K-wire group. Other studies reported average angulation in the cast group ranging from 14.6° (2–34°) to 30°, while the K-wire group ranged from 9.5° (0–18°) to 17°. Dorsal angulation in the K-wire group was as high as 28–32° in one study.

### 3.5. Quality Assessment

The quality of included studies was assessed using the Cochrane Risk-of-Bias tool for Randomized Trials (RoB 2) and the Methodological Index for Non-Randomized Studies (MINORS). All randomized controlled trials showed a high risk of bias, especially in performance and detection bias due to inadequate blinding. MINORS assessments revealed moderate methodological quality, with scores ranging from 8 to 12. Tables S3 and S4.

## 4. Discussion

Kirschner wire (K-wire) fixation is frequently used to stabilize displaced distal radius fractures, especially when closed reduction alone fails to provide adequate fracture alignment [26]. The method includes inserting wires through the skin to hold the fracture fragments in place, offering internal stabilization with minimal impact on the surrounding soft tissues [27]. After stabilizing the fracture with K-wires, a cast is usually applied to help preserve the alignment and promote proper healing [28]. This combined method is particularly effective for treating extra-articular fractures and those with limited fragmentation [29]. Research has consistently demonstrated that using K-wire fixation with casting yields good radiographic and functional outcomes over both the short and long term [11]. However, potential complications—such as infection at the pin site, displacement of the fracture, or migration of the wires—can arise, making precise surgical technique and diligent follow-up essential [30].

Our study showed a significantly higher re-displacement rate in the cast group (302/786) compared to the K-wire group (57/521), with an odds ratio of 11.42 (95% CI: 2.43–53.77, *p* = 0.002). Colaris et al. [19] who reported re-displacement of fracture of children treated with casting alone about 45%, versus only 8% with K-wire fixation. Similarly, findings by Miller et al. [17] observed a less re-displacement rate in the cast group compared to the pinning group, 39%: 0%, respectively [17]. These results suggest the use of K-wire fixation in unstable distal radius fractures to reduce the need for reintervention.

This study showed a significantly higher risk of secondary surgery in the cast immobilization group compared to K-wire fixation, OR 6.91 (95% CI: 1.5–31.72, *p* = 0.01). This indicates that casting alone often fails to maintain proper fracture, leading to the need for secondary procedures. McLauchlan et al. [22] observed that 7 out of 33 children treated with a cast needed further surgical correction, while no surgical correction in the K-wire group. Similarly, van Delft et al. [20] reported that 47% of children who received casting without fixation in the operating room needed secondary displacement, and 80% of these cases required further surgical treatment. The heterogeneity of this study was (I^2^ = 75%) reflecting varying practices and thresholds for reoperation, but in overall use of K-wire fixation to reduce the risk of secondary surgeries in unstable pediatric distal radius fractures.

About the comparing complication rates between cast immobilization and K-wire fixation, our study revealed that there is no significant relationship between the complication rates between the cast immobilization group and K-wire fixation group, OR: 0.68 (95% CI: 0.45–1.03, *p* = 0.07). This is like the results of Miller et al. [17], who reported a complication rate in the cast immobilization group compared with the K-wire group, with the results of 44% and 38%, respectively. However, the nature of the complications differed significantly [17]. These findings suggest that while complication rates may be similar, K-wire complications are generally less severe than cast-treated patients, which sometimes require surgical correction.

This study has several limitations. First, there was considerable heterogeneity among the included studies in terms of patient populations, surgical techniques, outcome measures, and follow-up durations, which may limit the generalizability and precision of the pooled estimates. Second, there was inconsistent reporting of complications and outcome measures across studies, making it challenging to standardize comparisons and assess clinical significance. Third, the lack of consistently reported Minimum Clinically Important Difference (MCID) values, particularly in the pediatric population, limited our ability to interpret whether statistically significant findings translated into meaningful clinical benefits. These factors highlight the need for more standardized and high-quality trials.

## 5. Conclusions

In conclusion, K-wire fixation appears to offer superior fracture stability and reduces the need for secondary surgeries compared to cast immobilization for displaced distal radius fractures in pediatric patients. However, both treatment modalities are associated with comparable complication rates, emphasizing the importance of individualized treatment planning. These findings contribute to the evidence base guiding the management of this common pediatric fracture and underscore the need for further research to optimize outcomes.

## Figures and Tables

**Table 1:** Basic demographics of the included studies.

Study ID	Year	Study Design	Country	Total Patients	Group 1 (Cast)	Group 2 (K-Wire)	Age Range (Years)	Males (n)	Females (n)
Wendling‚ ÄêKeim, 2015 [25]	2015	Retrospective	Germany	293	263	30	0–18		
Jordan, 2012 [24]	2012	Retrospective	United Kingdom	227	56	171	2–15	63	20
Diederix, 2021 [23]	2021	RCT	Netherlands	128	67	61	3–9		
McLauchlan, 2001 [22]	2001	RCT	Scotland	68	33	35	4–14	42	26
Ozcan, 2010 [21]	2010	Retrospective	Turkey	40	20	20	5–15 (K-wire), 6–14 (Cast)		
Delft, 2020 [20]	2020	Retrospective	Netherlands	200	175	25	8–14	118	82
Colaris, 2013 [19]	2012	RCT	Netherlands	128	67	61	<16	83	45
Gibbons, 1994 [18]	1994	Retrospective randomized	England	23	11	12	5–14	15	8
Miller, 2005 [17]	2005	Prospective randomized	United States	34	18	16	10–14	31	3
Pim W. Van Egmond, 2011 [16]	2011	Retrospective cohort	Netherlands	129	40	89	3–17	134	74
Wim van leemput, 2009 [15]	2009	Retrospective	Belgium	39	24	15	8–13	27	12
Joreg Schneider, 2007 [14]	2007	Retrospective	Switzerland	146	79	67	3–16	150	75

## Data Availability

The data generated in this study are available upon request from the corresponding author.

## Supplementary Materials

The following supporting information can be downloaded at: https://www.mdpi.com/article/10.3390/medicina61050852/s1, Table S1: PRISMA checklist. Table S2: Inclusion and Exclusion Criteria for Studies Included in the Systematic Review. Table S3: Risk of bias assessment according to Cochrane risk-of-bias tool for randomized trials (RoB 2); Table S4: Risk of bias assessment according to Methodological Index for Non-Randomized Studies (MINORS).

## Abbreviations

The following abbreviations are used in this manuscript:

DDRFs	displaced distal radius fractures.
MINORS	Methodological Index for Non-Randomized Studies
RCT	Randomized controlled study
CI	Confidence interval
