# Supplementary material for: Comparing the Outcomes of Cast Immobilization with and Without K-Wire Fixation for Displaced Distal Radius Fractures in the Pediatric Population: A Systematic Review and Meta-Analysis
Source: Medicina (Kaunas). 2025 May 6;61(5):852. doi: 10.3390/medicina61050852 (PMC12112781; doi:10.3390/medicina61050852)
Supplement: Supplementary file 1 [file medicina-61-00852-s001.zip › medicina-3564241-supplementary.pdf]

Section and Topic	Item #	Checklist item	Location where item is reported
**TITLE**			
Title	1	Identify the report as a systematic review.	1
**ABSTRACT**			
Abstract	2	See the PRISMA 2020 for Abstracts checklist.	2-3
**INTRODUCTION**			
Rationale	3	Describe the rationale for the review in the context of existing knowledge.	3
Objectives	4	Provide an explicit statement of the objective(s) or question(s) the review addresses.	3
**METHODS**			
Eligibility criteria	5	Specify the inclusion and exclusion criteria for the review and how studies were grouped for the syntheses.	3-4
Information sources	6	Specify all databases, registers, websites, organisations, reference lists and other sources searched or consulted to identify studies. Specify the date when each source was last searched or consulted.	4
Search strategy	7	Present the full search strategies for all databases, registers and websites, including any filters and limits used.	4
Selection process	8	Specify the methods used to decide whether a study met the inclusion criteria of the review, including how many reviewers screened each record and each report retrieved, whether they worked independently, and if applicable, details of automation tools used in the process.	4
Data collection process	9	Specify the methods used to collect data from reports, including how many reviewers collected data from each report, whether they worked independently, any processes for obtaining or confirming data from study investigators, and if applicable, details of automation tools used in the process.	4
Data items	10a	List and define all outcomes for which data were sought. Specify whether all results that were compatible with each outcome domain in each study were sought (e.g. for all measures, time points, analyses), and if not, the methods used to decide which results to collect.	4
	10b	List and define all other variables for which data were sought (e.g. participant and intervention characteristics, funding sources). Describe any assumptions made about any missing or unclear information.	4
Study risk of bias assessment	11	Specify the methods used to assess risk of bias in the included studies, including details of the tool(s) used, how many reviewers assessed each study and whether they worked independently, and if applicable, details of automation tools used in the process.	4
Effect measures	12	Specify for each outcome the effect measure(s) (e.g. risk ratio, mean difference) used in the synthesis or presentation of results.	4
Synthesis methods	13a	Describe the processes used to decide which studies were eligible for each synthesis (e.g. tabulating the study intervention characteristics and comparing against the planned groups for each synthesis (item #5)).	3-4
	13b	Describe any methods required to prepare the data for presentation or synthesis, such as handling of missing summary statistics, or data conversions.	3-4
	13c	Describe any methods used to tabulate or visually display results of individual studies and syntheses.	3-4

Section and Topic	Item #	Checklist item	Location where item is reported
	13d	Describe any methods used to synthesize results and provide a rationale for the choice(s). If meta-analysis was performed, describe the model(s), method(s) to identify the presence and extent of statistical heterogeneity, and software package(s) used.	3-4
	13e	Describe any methods used to explore possible causes of heterogeneity among study results (e.g. subgroup analysis, meta-regression).	3-4
	13f	Describe any sensitivity analyses conducted to assess robustness of the synthesized results.	3-4
Reporting bias assessment	14	Describe any methods used to assess risk of bias due to missing results in a synthesis (arising from reporting biases).	3-4
Certainty assessment	15	Describe any methods used to assess certainty (or confidence) in the body of evidence for an outcome.	3-4
**RESULTS**			
Study selection	16a	Describe the results of the search and selection process, from the number of records identified in the search to the number of studies included in the review, ideally using a flow diagram.	4
	16b	Cite studies that might appear to meet the inclusion criteria, but which were excluded, and explain why they were excluded.	4-5
Study characteristics	17	Cite each included study and present its characteristics.	4-5
Risk of bias in studies	18	Present assessments of risk of bias for each included study.	4-6
Results of individual studies	19	For all outcomes, present, for each study: (a) summary statistics for each group (where appropriate) and (b) an effect estimate and its precision (e.g. confidence/credible interval), ideally using structured tables or plots.	5-6
Results of syntheses	20a	For each synthesis, briefly summarise the characteristics and risk of bias among contributing studies.	5-6
	20b	Present results of all statistical syntheses conducted. If meta-analysis was done, present for each the summary estimate and its precision (e.g. confidence/credible interval) and measures of statistical heterogeneity. If comparing groups, describe the direction of the effect.	4-5
	20c	Present results of all investigations of possible causes of heterogeneity among study results.	4-5
	20d	Present results of all sensitivity analyses conducted to assess the robustness of the synthesized results.	4-6
Reporting biases	21	Present assessments of risk of bias due to missing results (arising from reporting biases) for each synthesis assessed.	5-6
Certainty of evidence	22	Present assessments of certainty (or confidence) in the body of evidence for each outcome assessed.	5
**DISCUSSION**			
Discussion	23a	Provide a general interpretation of the results in the context of other evidence.	9

Section and Topic	Item #	Checklist item	Location where item is reported
	23b	Discuss any limitations of the evidence included in the review.	9
	23c	Discuss any limitations of the review processes used.	10
	23d	Discuss implications of the results for practice, policy, and future research.	10
**OTHER INFORMATION**			
Registration and protocol	24a	Provide registration information for the review, including register name and registration number, or state that the review was not registered.	2
	24b	Indicate where the review protocol can be accessed, or state that a protocol was not prepared.	2
	24c	Describe and explain any amendments to information provided at registration or in the protocol.	2
Support	25	Describe sources of financial or non-financial support for the review, and the role of the funders or sponsors in the review.	11
Competing interests	26	Declare any competing interests of review authors.	11
Availability of data, code and other materials	27	Report which of the following are publicly available and where they can be found: template data collection forms; data extracted from included studies; data used for all analyses; analytic code; any other materials used in the review.	11

Table S1: PRISMA checklist.

	Inclusion Criteria	Exclusion Criteria
**Wendling,ÄêKeim, 2015**	Patients with displaced radial fractures aged 0,Äì18 years with epiphyseal plates	Salter/Harris 3-4 fractures, stress fractures, pathological fractures, refractures, bone diseases, pubertas praecox and tarda.
**Jordan, 2012**	Children under 16 years with operative intervention for distal third-radius fracture.	Open fractures, associated dislocations, and epiphyseal injuries excluded.
**Diederix, 2021**	Children with displaced forearm fracture stable after closed reduction.	Unstable fractures.
**McLauchlan, 2001**	Children with complete displaced distal radius fracture with or without ulnar fracture.	Physeal injury.
**Ozcan, 2010**	Distal metaphyseal radius fracture with high risk of re-displacement (translation > half bone diameter, angulation >30¬∞).	Patients do not meet inclusion criteria.
**Delft, 2020**	Patients under 16 years are treated for displaced distal forearm fractures.	Monteggia fractures, Galeazzi fractures, solitary ulna fractures.
**Colaris, 2012**	Children <16 years with displaced metaphyseal fracture of distal radius and ulna, stable after closed reduction.	Fractures older than 1-week, severe open fractures (Gustilo II-III), re-fractures.
**Gibbons, 1994**	Children under 15 with isolated distal radial fracture and complete displaced diaphyseal fracture of distal radius.	Incomplete, greenstick, or undisplaced distal radius fractures, displaced ulna fractures.
**Miller, 2005**	Children >10 years with skeletal immaturity and complete fracture of distal radius metaphysis with angulation >30¬∞.	Open fractures, prior injury or surgery, fractures requiring open reduction, neurovascular compromise.
**Pim W. Van Egmond, 2011**	Skeletal immature children with displaced extra-articular fractures and distal forearm.	Non-displaced distal forearm fractures not needing reduction, other fractures excluded.
**Wim van leemput, 2009**	NR	NR
**Joer Schneider, 2007**	Children with distal forearm fracture needing reduction or operation, x-rays reviewed.	Pathological fractures, patients initially treated at other facilities.

Table S2: Inclusion and Exclusion Criteria for Studies Included in the Systematic Review.

Authors/Year	Study Type	Selection Bias	Performance Bias	Detection Bias	Attrition Bias	Reporting Bias	Other Bias	Overall RoB
Diederix, 2021	RCT	Low	High	High	Unclear	Low	None	High
McLauchlan, 2001	RCT	Low	High	Unclear	Low	Unclear	None	High
Colaris, 2012	RCT	Low	High	Unclear	High	Low	None	High
Gibbons, 1994	RCT	Low	Unclear	Unclear	Unclear	Unclear	None	Unclear
Miller, 2005	RCT	Unclear	High	High	Low	Low	None	High

Table S3: Risk of bias assessment according to cochrane risk-of-bias tool for randomized trials (RoB 2)]

Authors/Year	1) A clearly stated aim	2) Inclusion of consecutive patients	3) Prospective collection of data	4) Endpoints appropriate to the aim of the study	5) Unbiased assessment of the study endpoint	6) Follow-up period appropriate to the aim of the study	7) Loss to follow-up less than 5%	8) Prospective calculation of the study size	9) An adequate control group	10) Contemporary groups	11) Baseline equivalence of groups	12) Adequate statistical analyses	Total Score
Wendling-Keim, 2015	2	1	0	2	0	1	0	0	1	2	0	1	10
Jordan, 2012	2	2	0	1	0	1	0	0	0	2	0	1	9
Ozcan, 2010	2	2	0	1	0	2	0	0	1	2	1	1	12
van Delft, 2020	2	2	0	1	0	2	0	0	1	2	1	1	12
van Egmond, 2012	2	2	0	1	0	2	0	0	1	2	0	1	11
VAN LEEMPUT, 2009	2	0	0	0	0	1	0	0	1	2	1	1	8
Schneider, 2007	2	2	0	1	0	1	0	0	0	2	0	1	9

Table S4: Risk of bias assessment according Methodological Index for Non-Randomized Studies (MINORS)
